# Preliminary evidence of an increased susceptibility to face pareidolia in postpartum women

**DOI:** 10.1098/rsbl.2023.0126

**Published:** 2023-09-13

**Authors:** Jessica Taubert, Samantha Wally, Barnaby J. Dixson

**Affiliations:** ^1^ School of Psychology, The University of Queensland, McElwain Building, St Lucia, 4072 Brisbane, Queensland, Australia; ^2^ Psychology and Social Sciences, The University of Sunshine Coast, Sippy Downs, Australia

**Keywords:** postpartum, face perception, face pareidolia, visual processing

## Abstract

As primates, we are hypersensitive to faces and face-like patterns in the visual environment, hence we often perceive illusory faces in otherwise inanimate objects, such as burnt pieces of toast and the surface of the moon. Although this phenomenon, known as face pareidolia, is a common experience, it is unknown whether our susceptibility to face pareidolia is static across our lifespan or what factors would cause it to change. Given the evidence that behaviour towards face stimuli is modulated by the neuropeptide oxytocin (OT), we reasoned that participants in stages of life associated with high levels of endogenous OT might be more susceptible to face pareidolia than participants in other stages of life. We tested this hypothesis by assessing pareidolia susceptibility in two groups of women; pregnant women (low endogenous OT) and postpartum women (high endogenous OT). We found evidence that postpartum women report seeing face pareidolia more easily than women who are currently pregnant. These data, collected online, suggest that our sensitivity to face-like patterns is not fixed and may change throughout adulthood, providing a crucial proof of concept that requires further research.

## 1. Background

**Figure 1.**
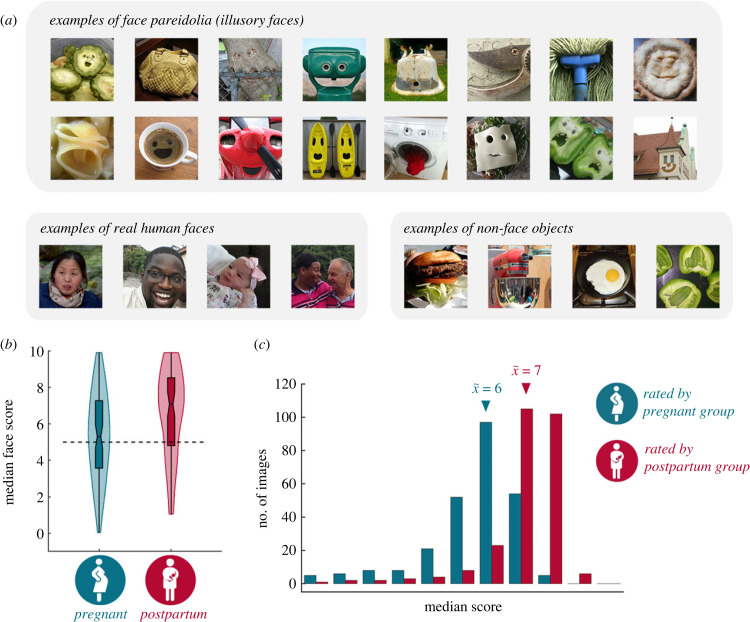
Postpartum women rate illusory faces as easier to see than pregnant women. (*a*) Illustrative examples of the experimental stimuli. (*b*) The violin plot visualizes the distribution of median face scores in the illusory face condition. The distributions are plotted separately for participants in the pregnant (*green*) and postpartum (*red*) groups. (*c*) To complement the participant-level analysis, here we plot the distribution of median scores for the 256 illusory faces (*green* = pregnant group, *red* = postpartum group). Arrows indicate grand median scores for both distributions.

As social primates, our wellbeing depends on our capacity to detect the faces of social agents. Thus, our sensitivity to faces and face-like patterns in the visual environment emerges immediately after birth [1–3], and is thought to underlie our propensity to perceive illusory faces in examples of face pareidolia [4–16] (figure 1*a*). But beyond the early stages of infancy, it is unclear whether our sensitivity to illusory faces changes across time. Although investigations have shown that susceptibility to face pareidolia varies across individual participants [17,18] and clinical populations [19–25] there has been no attempt to understand whether this susceptibility is stable across an individual's lifespan.

Oxytocin (OT) is a neuropeptide associated with maternal behaviors in mammals, including nursing, bonding, and prosocial behaviour during the postpartum period [26]. Interestingly, the administration of artificial OT has been found to increase sensitivity to various facial signals in primates [27,28] including humans [29] but these findings are constrained by questions surrounding the efficacy of artificial OT and peripheral administration methods [30,31]. Thus, to examine the link between face perception and OT, researchers have recruited women in the postpartum stage of childbirth [32–35] because that is when women experience their highest known levels of endogenous OT [36]. These studies have uncovered pronounced changes in the perception of facial attributes in postpartum women [37,38] but whether these changes include a greater sensitivity to face-like patterns remains unknown. Here, our goal was to determine whether postpartum women are more susceptible to face pareidolia. Using a robust self-report measure of pareidolia strength [11,16], we asked two groups of participants to view a large number of photographs and rate how easily they could see a face in each image. We expected that postpartum women would report seeing illusory faces in ordinary inanimate objects more easily than women in earlier stages of pregnancy.

## 2. Methods and methods

### (a) Participants

This experiment was approved by the University of Queensland's Human Research Ethics Committee. We targeted a sample size of 400 women and, consequently, a total of 401 participants were recruited via an online platform (https://www.prolific.co/). Participants had to complete more than 69% of the trials to be included in the final analysis. The 379 participants that met this criterion, self-identified as female (mean age = 28.3 years, s.d. = 7.3). We anticipated that the data from participants who did not indicate that they were pregnant, or in the post-partum stage of pregnancy, could be compared to data associated with the seminal study (see Exp. 1a ‘face ratings’ [16]). Importantly, although we were unable to acquire independent confirmation of pregnancy status, no information about the direction of the experimental hypotheses was given to the participants and thus they were unable to bias their responses.

### (b) Experimental stimuli

The images used as stimuli were identical to those used by Wardle *et al*. [16] and are available for download from the Open Science Framework. The images that were used as stimuli consisted of 32 images of human faces, 32 images of ordinary objects and 256 illusory faces (figure 1*a*).

### (c) Experimental procedure

The experimental procedure was originally designed by Wardle *et al*. [16] to measure face resemblance in examples of face pareidolia (see Exp. 1a [16]). The experiment was created using Qualtrics (Qualtrics XM, version 08.2022). Before the trials began, demographic information was collected, including pregnancy status. If a participant indicated they were currently pregnant, they were assigned to the pregnant group and asked to provide their due date. In contrast, if a participant indicated they had given birth in the past 12 months, they were assigned to the postpartum group, and asked to provide the date on which they gave birth.

Next the participants were presented with 320 trials with stimuli presented in a random order. When a trial began, the participant was presented with one of the images and asked ‘Do you see a face in the image below?’ To respond to the question, the participant was provided with an 11-point scale from 0 (No, I don't see a face) to 10 (Yes, I definitely see a face). The experiment was self-paced; the image with the instructions and the 11-point scale remained visible on the screen until a response was given. Once a response was given the next trial began. On average 320 trials took 36 min to complete. All data are available from a data repository: https://osf.io/6merf/ [39].

## 3. Results

The participants were sorted into three groups; those who were expecting to give birth (pregnant women; *n* = 84), those who had given birth in the last 12 months (postpartum women; *n* = 79) and those who indicated that they were neither pregnant nor in the postpartum period (control women; *n* = 216). All reported statistical tests are two-tailed. To confirm the successful replication of Wardle *et al*. [16], the average responses of the control women were compared to the average responses of the female participants in the original study (*N* = 394 [16]) using an independent samples *t-*test (*t*_608_ = 0.51, *p* = 0.61, Cohen's *d* = 0.04). However, since we did not survey biological metrics, such as menstrual cycle status, chronological age or genetics, participants in the control group were likely to vary greatly in terms of their OT levels [36]. Thus, the data from these participants were removed from the main analyses. Nonetheless, we used the control group data in the analysis of the *all groups design* where the expectation was that women in the pregnant group would give lower scores to illusory faces than those in the control group, whereas the women in the postpartum group would give higher scores to illusory faces than those in the control group.

### (a) Main analyses

We computed the median score given to illusory faces separately for the pregnant $\left(\tilde{x}=5.30,\;s.d.=2.31\right)$ and postpartum $\left(\tilde{x}=7.08,\;s.d.=2.38\right)$ participants (figure 1*b*). Remarkably, we found evidence that postpartum women reported being able to see face pareidolia more easily than pregnant women (independent samples Mann–Whitney *U*-test, *N* = 163, *U* = 3.46, *p* < 0.001). Concomitantly, we found no evidence of a difference in scores for real faces ($\tilde{x}\;(\mathrm{pregnant})\;=\;10,\;s.d.=0;\tilde{x}\;(\mathrm{postpartum})=10,\;s.d.=0;$ *N* = 163, *U* = 0, *p* = 1) or ordinary objects ($\tilde{x}\;(\mathrm{pregnant})=$ $0,s.d.=0.68;\tilde{x}\;(\mathrm{postpartum})=0,s.d.=0.77;$ *N* = 163, *U* = −0.61, *p* = 0.54).

Next, to determine whether the face scores for the illusory faces differed, we performed an image-based analysis and computed the median score given to each illusory face by each of the groups. For the purposes of this analysis, we changed the unit of analysis from ‘participant’ to ‘illusory face’ (figure 1*c*) and we treat the groups as conditions. As expected, we found that the illusory faces were rated as being more easily seen when they were rated by postpartum women $\left(\tilde{x}=7,s.d.=1.29\right)$ than when they were rated by pregnant woman ($\tilde{x}=6,s.d.=1.6$; related-samples Wilcoxon signed rank test, *N*_images_ = 256, *Z* = 14.02, *p* < 0.001; figure 1*c*).

### (b) All groups design

**Figure 2.**
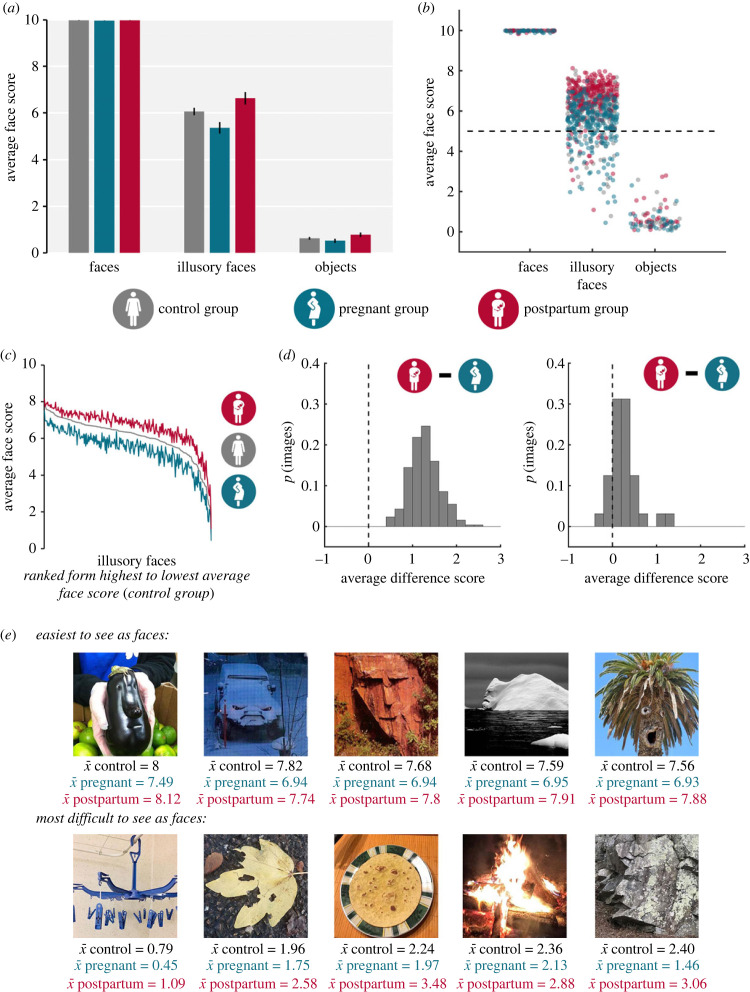
Analysis of variance with all three participant groups. (*a*) A bar graph showing average face scores as a function of stimulus condition with ‘participant’ as the unit of analysis. The averages for each group of participants are plotted separately (error bars indicate ± s.e.m.). (*b*) A bar graph showing the average face scores for each image (the partially transparent points with horizontal jitter set to 0.4 represent each image) as a function of stimulus condition. Colour indicates the participant group (*green* = the average score for an image based on 84 pregnant women, *red* = average score for an image based on 79 postpartum women, *grey* = average score for an image based on 216 control women). (*c*) The results of the rank-order analysis visualized as a line graph with illusory faces sorted on the *x*-axis by average face score (highest to lowest) according to the control group (*grey line*). When the same order was then applied to the data from the pregnant (*green line*) and postpartum groups (*red line*). The rank order correlation suggests that the relative scores for the illusory faces were consistent across groups, what changed was the magnitude of the face scores. (*d*) On the left is the distribution of average difference scores ($\bar{x}$ postpartum – $\bar{x}$ pregnant) for every stimulus in the pareidolia condition. The median value in this distribution is 1.26. The range of difference scores goes from 0.51 to 2.47 indicating that, on average, *all* illusory faces were rated higher, and more easily seen as faces, by women in the postpartum group than those in the pregnant group. On the right is the distribution of difference scores for the stimuli in the object condition (*N* = 32, median difference score = 0.21, minimum difference score = −0.21, maximum difference score = 1.27). (*e*) Average face scores for 10 representative pareidolia images. *Top row:* the five most easily seen illusory faces, with the highest average face scores according to the control group. *Bottom row:* the five most difficult to see illusory faces, with the lowest average face scores according to the control group.

Similar to previous studies of face pareidolia using face resemblance scores, we employed a fixed effects factorial ANOVA to compare the effect of stimulus condition across the three groups [7,13,24,40]. However, we note that this analysis requires us to treat the ordinal data as continuous [41]. To this end, we first averaged the trial data such that every participant had an average face, illusory face and object score. Then, using a 3 × 3 ANOVA with stimulus condition (faces, illusory faces, and objects) as a repeated factor and participant group (control, pregnant, and postpartum) as a between-groups factor, we found the expected main effects of stimulus condition (*F*_2,752_ = 4036.52, *p* < 0.001, *η*_p_*^2^* = 0.91) and participant group (*F*_2,376_ = 6.09, *p* = 0.002, *η*_p_*^2^* = 0.03). Note that figure 2 shows average scores, not median scores as in figure 1*b*, because ANOVA is an analysis of means. The significant interaction (*F*_4,752_ = 5.5, *p* < 0.001, *η*_p_*^2^* = 0.03) was followed by nine discrete pairwise comparisons using a critical *p*-value adjusted for multiple comparisons (0.05/9). These revealed only one significant difference: women in the postpartum condition gave higher scores to illusory faces than pregnant women (*t*_161_ = 3.46, *p* < 0.001, Cohen's *d* = 2.34). All other *p*-values ranged from 0.02 to 0.91 and did not survive the Bonferroni adjustment (figure 2*a*). This included all comparisons between groups for the real face and object conditions. These observations indicate that the differences between pregnant women and postpartum women were unique to the illusory face condition, although future research needs to rule out more general perceptual and cognitive biases.

Importantly, the stimuli in the real face condition were all rated as being easily seen as faces (range of average ‘face scores’ for real faces when rated by control women = 9.94–10; range of average ‘face scores’ for real faces when rated by pregnant women = 9.88–10; range of average ‘face scores’ for real faces when rated by postpartum women = 9.83–10). These findings suggest that the participants understood the task instructions and rated all 32 real human faces appropriately.

### (c) Rank-order analysis

To determine the consistency with which illusory faces were rated across the three groups of participants, we performed a rank-order analysis. All 256 illusory faces were sorted from the example with the highest average score to the example with the lowest average score based on the responses of the control participants. This ranking was then applied to the data collected from pregnant and postpartum women (figure 2*c*). Pairwise comparisons of the groups revealed significant rank-order correlations (Spearman's *ρ* tests; *control* versus *pregnant*, ρ=0.95,p<0.001; *control* versus *postpartum*, ρ=0.96,p<0.001; *pregnant* versus *postpartum*, ρ=0.94,p<0.001).

To examine this further, we calculated the difference score for each illusory face by subtracting the average face score in the pregnant group from the average face score in the postpartum group (figure 2*d*). The distribution of difference scores reveals that *all* illusory faces were given higher scores by postpartum women compared to pregnant women ($\tilde{x}=1.26$; one-sample Wilcoxon signed rank test with a hypothetical median = 0, *N*_images_ = 256, *Z* = 13.87, *p* < 0.001; figure 2*d*). For comparison, difference scores were also calculated for the ordinary objects ($\tilde{x}=0.21,$ *N*_images_ = 32; figure 2*d*) and we found that the illusory faces elicited larger differences from the two target groups than the objects (independent samples Mann–Whitney *U*-test, *N* = 288, *U* = 8.71, *p* < 0.001). The highest and lowest scoring illusory faces are provided in figure 2*e*.

## 4. Discussion

Our data, collected online, indicate that postpartum women report seeing illusory faces in examples of face pareidolia more easily than pregnant women, suggesting that our sensitivity to face pareidolia changes during adulthood. Self-reported experiences of face pareidolia have been linked to dementia [20], Parkinson's disease [21,23,42], and increased feelings of loneliness [43], making it a valuable clinical tool. Our findings contribute to this growing literature by suggesting that our sensitivity to face pareidolia is heightened during early parenthood, possibly promoting social bonding, which is thought to be critical in maternal–infant dyads [44].

Importantly, our findings also suggest that endogenous OT might contribute to changes in our sensitivity to faces and capacity for face detection. There is a large body of research showing that the administration of OT to humans and non-human primates increases attention towards faces and facial signals [27–29,45]. Our findings are consistent with this view, showing that human participants with naturally higher levels of OT, report seeing illusory faces more easily than participants with naturally lower levels of OT. However, since we were unable to measure the level of endogenous OT in our participants, the link between susceptibility to face pareidolia and OT requires further investigation.

In sum, we have unearthed preliminary evidence that postpartum women are more susceptible to face pareidolia than pregnant women. We targeted these two groups because they are known to differ in terms of OT levels, however, we did not measure their OT levels and thus it is possible that other differences between these groups explain our results. For example, it could be that differences in anxiety or stress account for the observed differences. Nonetheless, the observation that these two groups reliably differed in their susceptibility to face pareidolia provides that first demonstration that our sensitivity to faces is not stable throughout our adult lives. Quantifying the factors that predict changes in this sensitivity will be essential for advancing our neural models of face perception and for deciding whether face pareidolia is just a fun side effect of a hypersensitivity to faces that we share with other primates [4,46], or a diagnostic tool that could be leveraged to monitor disease progression and flag mental health decline [20,21,23,25].

## Data Availability

All data and research materials supporting the results in this article are available in an external data repository in the format collected from Qualtrics (raw numerical values per trial with no preprocessing): https://osf.io/6merf/ [39]. Data were analysed using two software packages, Matlab verison R2021a (www.mathwork.com) and SPSS (https://www.ibm.com/products/spss-statistics).
